# Increases in regional brain volume across two native South American male populations

**DOI:** 10.1007/s11357-024-01168-2

**Published:** 2024-04-29

**Authors:** Nikhil N. Chaudhari, Phoebe E. Imms, Nahian F. Chowdhury, Margaret Gatz, Benjamin C. Trumble, Wendy J. Mack, E. Meng Law, M. Linda Sutherland, James D. Sutherland, Christopher J. Rowan, L. Samuel Wann, Adel H. Allam, Randall C. Thompson, David E. Michalik, Michael Miyamoto, Guido Lombardi, Daniel K. Cummings, Edmond Seabright, Sarah Alami, Angela R. Garcia, Daniel E. Rodriguez, Raul Quispe Gutierrez, Adrian J. Copajira, Paul L. Hooper, Kenneth H. Buetow, Jonathan Stieglitz, Michael D. Gurven, Gregory S. Thomas, Hillard S. Kaplan, Caleb E. Finch, Andrei Irimia

**Affiliations:** 1 Department of Biomedical Engineering, Viterbi School of Engineering, University of Southern California, Los Angeles, CA USA; 2 Ethel Percy Andrus Gerontology Center, Leonard Davis School of Gerontology, University of Southern California, Los Angeles, CA USA; 3 Center for Economic and Social Research, Dana and David Dornsife College of Letters, Arts and Sciences, University of Southern California, Los Angeles, CA USA; 4 Center for Evolution & Medicine, School of Human Evolution and Social Change, School of Life Sciences, Arizona State University, Tempe, AZ USA; 5 Department of Population and Public Health Sciences, Keck School of Medicine, University of Southern California, Los Angeles, CA USA; 6 iBRAIN Research Laboratory, Departments of Neuroscience, Computer Systems and Electrical Engineering, Monash University, Melbourne, VIC Australia; 7 Department of Radiology, The Alfred Health Hospital, Melbourne, VIC Australia; 8 Department of Neurology, Keck School of Medicine of USC, University of Southern California, Los Angeles, CA USA; 9 MemorialCare Health Systems, Fountain Valley, CA USA; 10 Renown Institute for Heart and Vascular Health, Reno, NV USA; 11 School of Medicine, University of Nevada, Reno, NV USA; 12 Division of Cardiology, University of New Mexico, Albuquerque, NM USA; 13 Department of Cardiology, School of Medicine, Al-Azhar University, Al Mikhaym Al Daem, Cairo, Egypt; 14 Saint Luke’s Mid America Heart Institute, University of Missouri, Kansas City, MO USA; 15 Department of Pediatrics, School of Medicine, University of California, Irvine, Orange, CA USA; 16 MemorialCare Miller Children’s & Women’s Hospital, Long Beach Medical Center, Long Beach, CA USA; 17 Division of Cardiology, Mission Heritage Medical Group, Providence Health, Mission Viejo, CA USA; 18 Universidad Peruana Cayetano Heredia, Lima, Peru; 19 Department of Anthropology, University of New Mexico, Albuquerque, NM USA; 20 Economic Science Institute, Argyros School of Business and Economics, Chapman University, Orange, CA USA; 21 Department of Anthropology, University of California, Santa Barbara, USA; 22 Institute of Biomedical Research, San Simon University, Cochabamba, Bolivia; 23 Tsimane Health and Life History Project, San Borja, Beni, Bolivia; 24 Institute for Advanced Study in Toulouse, Toulouse 1 Capitol University, Toulouse, France; 25 Division of Cardiology, University of California, Irvine, Orange, CA USA; 26 Departments of Biological Sciences, Anthropology and Psychology, Dana and David Dornsife College of Letters, Arts and Sciences, University of Southern California, Los Angeles, CA USA; 27 Department of Quantitative and Computational Biology, Dana and David Dornsife College of Letters, Arts and Sciences, University of Southern California, Los Angeles, CA USA

**Keywords:** Brain aging, Neurodegeneration, Cortex

## Abstract

Industrialized environments, despite benefits such as higher levels of formal education and lower rates of infections, can also have pernicious impacts upon brain atrophy. Partly for this reason, comparing age-related brain volume trajectories between industrialized and non-industrialized populations can help to suggest lifestyle correlates of brain health. The Tsimane, indigenous to the Bolivian Amazon, derive their subsistence from foraging and horticulture and are physically active. The Moseten, a mixed-ethnicity farming population, are physically active but less than the Tsimane. Within both populations (*N *= 1024; age range = 46–83), we calculated regional brain volumes from computed tomography and compared their cross-sectional trends with age to those of UK Biobank (UKBB) participants (*N* = 19,973; same age range). Surprisingly among Tsimane and Moseten (T/M) males, some parietal and occipital structures mediating visuospatial abilities exhibit small but significant increases in regional volume with age. UKBB males exhibit a steeper negative trend of regional volume with age in frontal and temporal structures compared to T/M males. However, T/M females exhibit significantly steeper rates of brain volume decrease with age compared to UKBB females, particularly for some cerebro-cortical structures (e.g., left subparietal cortex). Across the three populations, observed trends exhibit no interhemispheric asymmetry. In conclusion, the age-related rate of regional brain volume change may differ by lifestyle and sex. The lack of brain volume reduction with age is not known to exist in other human population, highlighting the putative role of lifestyle in constraining regional brain atrophy and promoting elements of non-industrialized lifestyle like higher physical activity.

**Supplementary Information:**

The online version contains supplementary material available at 10.1007/s11357-024-01168-2.

## Introduction

Aging-related brain atrophy has been studied extensively among populations in industrialized countries such as the US [1–14], UK [2, 9], Japan [7, 11], Switzerland [12], and the Netherlands [13]. The extent of age-related atrophy varies across the cortex [15], according to both longitudinal [2, 16, 17] and cross-sectional studies [18]. The frontal and temporal lobes, striatum, cerebellum, and hippocampus are thought to atrophy faster, on average, than the rest of the brain [19]. In males, frontal and temporal cortices atrophy fastest; in females, the hippocampi and parietal lobes decrease fastest with age [4, 20]. Monitoring the spatial and temporal profiles of brain atrophy can be useful when assessing the risk of neurodegenerative diseases including Alzheimer’s disease. For example, Alzheimer’s disease progression is paralleled by faster-than-normal regional brain atrophy, first observed in mesial temporal structures (i.e., entorhinal and hippocampal formations), then in the posterior temporal and parietal lobes, and finally in frontal brain structures [21, 22].

Although frequently linked to better healthcare and sanitation, industrialization also involves adverse lifestyle factors such as sedentarism and heavy consumption of processed foods. This may partly explain observed variability in brain atrophy trajectories [23, 24], health outcomes [25], and dementia rates [26] across industrialized and certain non-industrialized populations. Partly for this reason, studying the age dependence of brain volume across such populations can help to understand its correlates with brain health. The Tsimane (population $\sim$ 17,000) are an indigenous South American population of forager-horticulturists whose subsistence lifestyle in the Bolivian Amazon, until recently, involved minimal interaction with the broader Bolivian population [27]. Neighboring the Tsimane are the Moseten (population $\sim$ 3000), who are genetically and culturally related to the Tsimane but more acculturated to the Bolivian population [28]. Unlike the Tsimane, the Moseten have access to modern amenities such as running water, electricity, sanitation, medical services, and market foods [27, 29, 30].

Compared to their industrialized counterparts, both the Tsimane and Moseten (T/M) have fewer dementia risk factors (e.g., cardiovascular disease, type 2 diabetes, smoking), are more physically active, and consume more fish, fruit, and vegetables [24, 25]. Differences in industrialization exposure between the UK—on the one hand—and the T/M—on the other hand—highlight the utility of comparing these populations to those in the industrialized world. In industrialized populations, physical activity and diets rich in fiber and healthy fats are typically associated with reduced brain atrophy [31, 32]. The T/M have high levels of both physical activity [29] and systemic inflammation [33, 34]. The latter accelerates brain atrophy in industrialized populations [35–37]. Nevertheless, in the Tsimane, the cross-sectional relationship between total brain volume and age is significantly shallower than in certain industrialized samples from the Netherlands, US, and Germany [23]. The current study quantifies the dependence of 148 cortical gray matter (GM) *regional* volumes on age in T/M and compares these age-related associations to those of adults in the UK according to sex. Across industrialized and non-industrialized societies, our findings help to contextualize lifestyle’s role in constraining regional age-related brain atrophy rates, which parallel dementia risk.

## Methods

### Participants

Participants (Table 1) included 746 Tsimane, 434 Moseten, and 19,973 adults from the UK Biobank (UKBB, https://www.ukbiobank.ac.uk/). Ethical approval was obtained from the Institutional Review Board of University of California Santa Barbara (IRB #15–133), Universidad Mayor San Simon, Cochabamba Bolivia, and from the local ethical boards of all other institutions where research was performed. Ethical approval was also obtained from indigenous governments (Gran Consejo Tsimane, Consejo Regional Tsimane y Moseten, Organización del Pueblo Indigena Moseten), from community leaders, and from all study participants. UKBB data were acquired with ethical approval from the North-West Multi-Centre Research Ethics Committee of the United Kingdom [38]. UKBB participants exhibit a healthy volunteer selection bias because, compared to the general UK population, they live in less socioeconomically deprived areas, are less likely to be obese, to smoke, to drink alcohol, and to have self-reported health conditions [39]. In 13 U.S. adults aged 55 to 75, both CTs and MRI scans were acquired for validation.

**Table 1:** Sample demographics

Repository	*N*	*μ* (age)	*σ* (age)	Males:Females
Tsimane	673	60.2	9.2	1:0.83
Moseten	351	58.0	8.8	1:0.73
UK Biobank	19973	64.4	7.7	1:1.10

### Imaging

For UKBB participants, ${T}_{1}$-weighted magnetic resonance imaging (MRI) scans were acquired at 3 T using Siemens Skyra MRI scanners (software platform VD13, 32-channel receiving head coil, 3D acquisition, magnetization-prepared rapid gradient echo sequence, voxel size = 1.0 mm  ×  1.0 mm  ×  1.0 mm, matrix size = 208  ×  256  ×  256, inversion time [TI] = 800 ms, repetition time [TR] = 2 s, in-plane acceleration factor = 2). For protocol details, see Alfaro-Almagro, Jenkinson [40]. These scans were acquired between 2014 and 2019 [41].

For T/M participants, CT scans were acquired using a 16-detector row scanner (General Electric BrightSpeed, Milwaukee, WI). Images were acquired clockwise, in helical mode, with a standard convolution kernel, and two reconstructions: one with a voxel size of 1.25 mm  ×  1.25 mm  ×  1.25 mm and another with a voxel size of 0.625 mm  ×  0.625 mm  ×  0.625 mm. Additional parameters include a kilovoltage peak of 120 kV, a data collection diameter of 25 cm, a mean exposure time of 1.417 s, an X-ray tube current of 140 mA, and a focal spot of 0.7 mm. These scans were acquired between 2015 and 2018.

### Imaging for CT/MRI validation

CT scans used for segmentation validation were acquired using a Toshiba Aquilion ONE scanner and had scan parameters akin to those of T/M scans. Images were acquired clockwise, in helical mode, with a Toshiba FC68 convolution kernel and a voxel size of 0.46 mm  ×  0.46 mm  ×  0.60 mm. Additional parameters included a kilovoltage peak of 120 kV, a data collection diameter of 32 cm, a mean exposure time of 1 s, an X-ray tube current of 140 mA, and a focal spot of 0.8 mm.

${T}_{1}$-weighted MRIs used for validation were acquired at 3 T using a Prisma MAGNETOM Trio TIM scanner (Siemens Corp., Erlangen, Germany), a magnetization-prepared rapid acquisition gradient echo sequence, and the following parameters: TR = 1.950 s; echo time = 3 ms; TI = 900 ms; flip angle = 9 degrees; percentage sampling = 100; pixel bandwidth = 240 Hz/pixel; matrix size = 256 × 256; voxel size = 1 mm × 1 mm × 1 mm.

### Image processing

The recon-all function of Freesurfer (FS) software version 6.0 [42] was used to segment validation MRIs according to the Destrieux cortical parcellation scheme [43]. The naming convention for brain structures studied here is that of the FS parcellation scheme. For example, the term *sulcus* refers here to the gray matter (GM) tissue associated with the trough (invagination) of the cerebral cortex, rather than to the extracerebral space within the sulcal groove itself, which contains cerebrospinal fluid. The UKBB repository provides FS processed and segmented files, which were used to extract regional volumes.

CT scans were segmented using a two-step approach. First, based on voxel intensity values, a probabilistic classification algorithm was used to segment the brain into one of five tissue classes: GM, white matter, cerebrospinal fluid, scalp, and skull, as described elsewhere [14]. Second, an algorithm was used to segment cortical GM into gyral and sulcal structures according to the Destrieux parcellation scheme. The GM probability map was binarized and a spatial bias function was used to correct the radiodensity gradient along the inferior-superior axis of the CT volume. Next, three successive linear transformations (rigid, similarity, and affine) were applied iteratively to register the cortical GM of the FS atlas to each participant’s GM mask. A final nonlinear registration improved registration quality. GM voxels were labeled according to the Destrieux parcellation scheme.

### CT/MRI validation

Let $r=1,\dots ,R$ be a cortical structure in the FS Destrieux parcellation scheme, where $R=148$ is the number of structures, and let $N$ be the validation sample size of 13. Let ${w}_{r}$ denote the percentage of cortical GM volume accounted for by $r$ and let ${v}_{ir}^{CT}$ and ${v}_{ir}^{MRI}$ be the volumes of $r$ for subject $i$, as derived from CT and MRI, respectively. The average difference ${\Delta v}_{r}$ between ${v}_{ir}^{CT}$ and ${v}_{ir}^{MRI}$ was computed as ${\Delta v}_{r}\text{ = }\frac{1}{{\text{N}}}{\sum\limits_{{\text{i}}= \text{1} }^{\text{N}}}\left({v}_{ir}^{CT}- \, {v}_{ir}^{MRI}\right)$. The weighted average $\varpi =\frac{1}{R} {\sum \limits_{r=1}^{R}{w}_{r}}\left|{\Delta v}_{r}\right|$ of absolute differences in volumes was used to measure the discrepancy between CT and MRI volumes.

### Volumetric normalization

To ease interpretation, two normalizations were applied to the regional brain volumes ${v}_{ir}$, for regions $r=1,\dots ,\,R$ in subject *i* of each population. In the first of these, to account for variation in head sizes, each ${v}_{ir}$ was divided by the intracranial volume ${v}_{i}^{ICV}$, yielding ${v}^{{}\, \prime}_{ir}\equiv {v}_{ir}/{v}_{i}^{ICV}$. For each $r$, a linear regression coefficient ${\beta }_{r}$ was then calculated to express the ICV-normalized regional volume vector ${{\varvec{V}}}_{r}{\prime}=[{v}_{1r}{\prime},\dots ,{v}_{ir}{\prime},\dots ,{v}_{Nr}{\prime}{]}^{T}$ as a function of the participants’ ages ${\varvec{A}}=[{a}_{1}{\prime},\dots ,{a}_{i}{\prime},\dots ,{a}_{N}{\prime}{]}^{T}$ using the equation ${{\varvec{V}}}_{r}{\prime}\, ={\beta }_{r}{\varvec{A}}$ + ${c}_{r}$, where ${c}_{r}$ is the intercept. For example, ${{v}_{r}{\prime}\, \left(46\right)=\beta }_{r}\times 46 y$ + ${c}_{r}$ is the average brain volume predicted by the regression equation for an individual with the youngest age in our sample, i.e., 46 y (years). For the second normalization, each volume ${v}_{ir}{\prime}$ was divided by ${v}_{r}{\prime}\, \left(46\right)$, yielding ${v}_{ir}^{{\prime}{\prime}}=100 \times {v}_{ir}{\prime}/{v}_{r}{\prime}\, \left(46\right)$. By adjusting volumes in this way for their regression-predicted average volume at age 46, one can express average cross-sectional decreases in volume after age 46 as a percentage of the expected volume at the sample’s youngest age (46 y). The values of ${v}_\mathit{ir}^{{\prime}{\prime}}$ were used in all subsequent analyses.

### Age-related regional volume trajectories

After normalization, linear regressions were used to examine regional volumes’ relationships to age in each population. Let $\beta$ be the regression coefficient denoting the cross-sectional annual rate of change in regional volume, after adjustment for head size and for the regression-predicted mean volume at the youngest age of 46 y. With these normalizations, a negative $\beta$ indicates that regional volume decreases at a rate of $\beta$%/year relative to the initial age of 46 y, when initial regional volume is 100%. Regression coefficients describing how regional volumes trend with age were calculated for Tsimane (${\beta }_{T}$), Moseten (${\beta }_{M}$), and UKBB (${\beta }_{UK}$) participants. Prior to regression, normalized brain volumes and age were converted to standardized $z$-scores using the means and standard deviations of each population and sex. Thus, regressions produced standardized regression coefficients ${\beta }_{s}$ that facilitated direct comparison between groups’ age-related effects on regional volume (negligible, ${\beta }_{S}<0.10$; small, $0.10\le {\beta }_{S}<0.30$; medium, $0.30\le {\beta }_{S}<0.50$; large, ${\beta }_{S}\ge 0.50$) [44].

95% confidence intervals for ${\beta }_{T}$, ${\beta }_{M}$, and ${\beta }_{UK}$ were obtained through bootstrapping, which was implemented separately for males and females within each cohort. One thousand random subsamples of size 100 were drawn from the Tsimane/Moseten samples (male or female). An age- and sex-matched subsample of size 100 was drawn from the UKBB sample. $\beta$’s were calculated at every realization. After 1000 realizations, $\mu \left(\beta \right)$ and $\sigma \left(\beta \right)$ were computed over all realizations. To examine the effects of age on normalized brain volume, the null hypothesis ${H}_{0 }:\beta = 0$ was tested at a significance threshold $\alpha = 0.05$ for each region’s$\beta$. Given that there are 148 regions, Bonferroni corrections with $\alpha = 0.05/148$ were implemented for multiple comparisons. To assess laterality effects, the age-related trends of regional volumes were compared between the left and right hemispheres. Similarly to regional volumes, the age-related rate of total brain, GM, WM, and cortical GM volume change was calculated for each population and sex.

### Comparison between Tsimane/Moseten and UKBB

For each brain structure, Welch’s two-tailed $t$-test for independent samples with unequal variances was used to test the null hypotheses ${H}_{0 }:{\beta }_{T}= {\beta }_{UK}$ and ${H}_{0 }:{\beta }_{M}= {\beta }_{UK}$ at a significance threshold $\alpha$ = 0.05/148 after Bonferroni corrections. To quantify differences in cross-sectional rate of cortical volume change, the quantity $\kappa =100\times {\sum }_{r=1}^{R}m(r){w}_{r}$ was computed across cortical regions whose regression coefficients differed significantly between Tsimane and UKBB. If the null hypothesis ${H}_{0 }:{\beta }_{T}= {\beta }_{UK}$ was rejected because ${\beta }_{T}< {\beta }_{UK}$, $m(r)$ was assigned the value 1, indicating that regional volume decrease was faster in Tsimane compared to the UKBB. To indicate the reverse, if the same null hypothesis was rejected because ${\beta }_{T}> {\beta }_{UK}$, then $m(r)$ was assigned the value − 1. If the null hypothesis could not be rejected, $m\left(r\right)=0$. An analogous procedure was used to compare ${\beta }_{M}$ to ${\beta }_{UK}$. If $\kappa >0$ then, on average, cortical GM volume decreases faster in the T/M than in the UKBB. If $\kappa <0$ then, on average, cortical GM volume decreases faster in the UKBB than in the T/M.

### Cognitive ability and physical activity

After regional volume comparison, visuospatial abilities were assessed in T/M using the stick design test. This test by Baiyewu et al. [45] is culturally agnostic and consists of reconstructing the printed designs of four models using four matchsticks. Test scoring is based on variations in configuration, orientation of the whole figure, and orientation of the matchsticks. Standardized stick scores and ages were computed as the $z$-scores of stick design test scores and ages, respectively, within each sex and population. In the T/M, the average interval between CT scan acquisition and the administration of the stick design test was 145 days (95% CI = [130, 160] days, median = 61 days). Stick design test scores were not available for the UKBB.

The number of minutes of moderate physical activity across 24 h was measured using wrist-worn ActiGraph GT3X accelerometers. Thresholds separating moderate activity from light and vigorous activity were determined to have a classification accuracy of 87% in laboratory settings [46, 47]. In T/M, the average interval between the CT scan and accelerometry data acquisition was 1377 days (95% CI = [1338, 1417] days, median = 1397 days). Accelerometry data were not available for the UKBB. We tested the hypothesis that cognitive ability and physical activity mediate the relationships between regional brain volumes and age. To this end, single-level mediation analysis examined whether visuospatial ability (stick scores) or physical activity (minutes of moderate activity per day) mediated the relationship between age and brain volume within each sex and population. We used the MediationToolbox (v1.0.0) in MATLAB (mediation.m available at https://github.com/canlab/MediationToolbox). The regression coefficient ${\beta }_{a}$ from a univariate linear regression captured the direct effect between standardized age (independent variable) and the volume of interest (dependent variable). Then, the mediator was included in a bivariate linear regression to estimate the indirect effect of age ${\beta }^{\, \prime}_{a}$ on brain volume and the mediator’s effect on brain volume ${\beta }^{\, \prime}_{t}$. The effect of age on the mediator ${\beta }_{b}$ was also estimated in a third, separate linear regression. Statistical inference of the mediation effect was made by calculating the product of (A) the age-adjusted mediator association with volume ${\beta }^{\, \prime}_{t}$ and (B) the effect of age on the mediator ${\beta }_{b}$ to a *t*-statistic. Bootstrapping (1000 iterations) was used to obtain an empiric distribution of this product mediation effect and allow statistical inference.

## Results

### Quality assessment and CT/MRI validation

Because acquiring MRI scans from T/M was not feasible, CT scans were acquired instead. Regional brain volumes were extracted from these scans using an automatic CT segmentation technique validated on a sample with both CT and MRI scans. In the CT/MRI validation sample, regional volumes derived from CT differed from those based on MRI by an average of 2.5% of the MRI (gold standard) volume ($\varpi =2.5$, see “Methods”). Segmentation quality was examined in 1180 T/M CT scans. Seven subjects were removed due to incorrigible segmentation errors. From among the remaining scans, 1024 were selected to match the age range (46–83 years old) of UKBB participants (Table 1).

### Regional brain atrophy

**Fig. 1:**
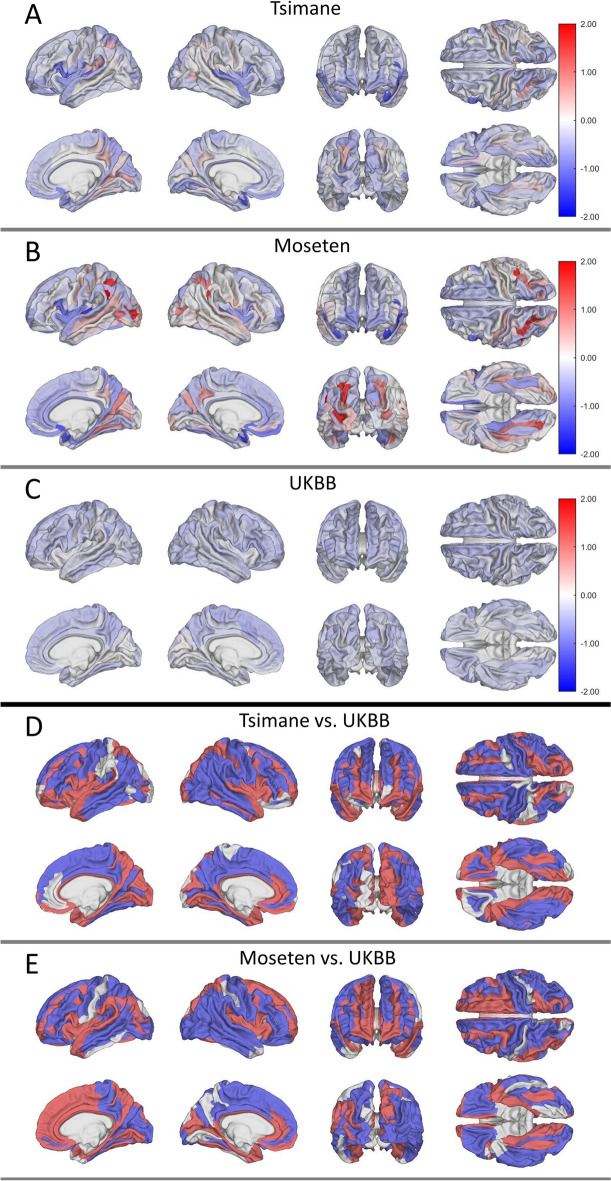
Regression coefficients for the association between cortical structures and age in males for Tsimane (**A**), Moseten (**B**), and Britons in the UK Biobank (**C**). Brighter color indicates a larger magnitude of the regression coefficient for the corresponding structures. **D**, **E** Structures whose regression coefficients differ significantly from the UKBB in Tsimane (**D**) and Moseten (**E**). Blue indicates faster volume decrease in the UKBB compared to the Tsimane/Moseten. Red indicates faster volume decrease in the Tsimane/Moseten compared to the UKBB. On average, 54% of the cortex exhibits faster decrease with age in the UKBB compared to the Tsimane and 51% of the cortex exhibits faster decrease with age in the UKBB compared to the Moseten

**Fig. 2:**
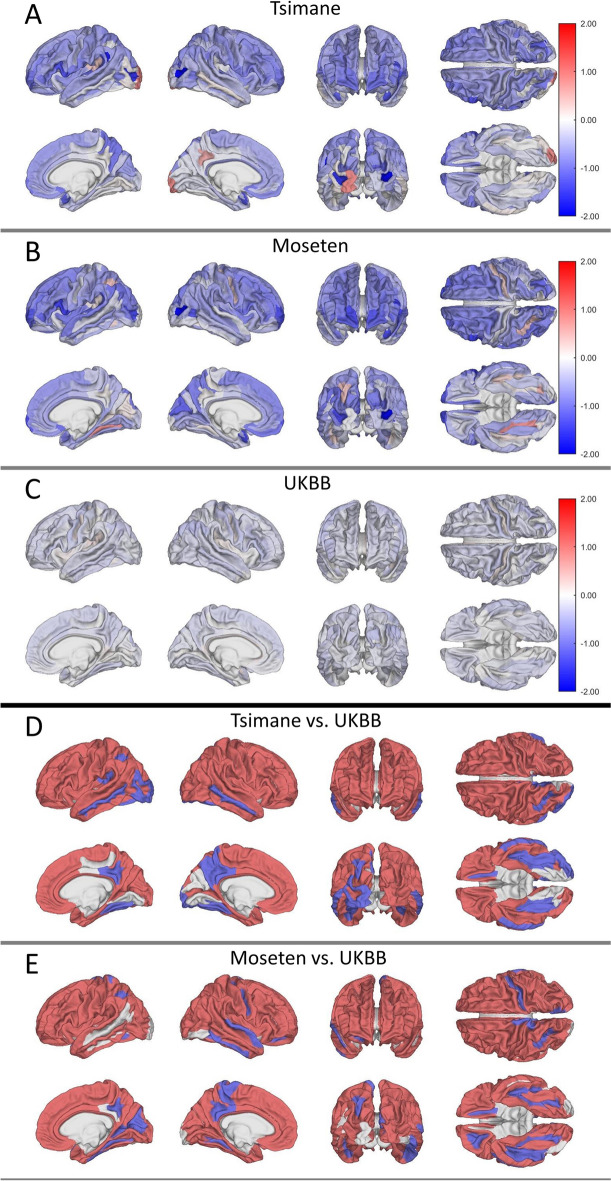
As in Fig. 1, but for females. On average, 82% of the cortex exhibits faster atrophy in the Tsimane compared to UKBB; 84% of the cortex exhibits faster atrophy in the Moseten compared to UKBB

Across both sexes, 82% of regression coefficients $\beta$ are negative in T/M, indicating cross-sectional decreases in normalized cortical volume with age across 82% of structures. We denote unstandardized regression coefficient for the Tsimane by ${\beta }_{T}$, Moseten by ${\beta }_{M}$, and UKBB by ${\beta }_{UK}$. To allow comparison between the samples, we also compute standardized regression coefficient, represented with ${\beta }_{s}$. Medium effect sizes (${\beta }_{s }>0.3$) of age on regional brain volume are found among only 13 structures ($\sim$ 10%) in males and 33 ($\sim$ 20%) in females, while remaining structures exhibit smaller effect sizes (${\beta }_{s }<0.3$). Supplementary Material 1 provides a complete list of regression coefficients and effect sizes; Table 2 lists 27 structures with the largest effect sizes (${\beta }_{s }>0.4$). In Tsimane males, 111 of 148 structures’ volumes (75% of structures, 80% of the cortex) trend negatively with age (Fig. 1A), while 134 of 148 Tsimane female brain structures (91% of structures, 90% of the cortex) exhibit cross-sectional decline with age (Fig. 2A). Similarly, in Moseten males (Fig. 1B), 89 of 148 structures (60% of structures, 66% of the cortical gray matter) exhibit a negative trend of volume with age. Of these, the left planum polare of the superior temporal gyrus exhibits the largest effect size, decreasing in cross-section at a rate of 1.38% per year of age ($p$< 0.001, ${\beta }_{s}$=  − 0.52). In Moseten females, 130 of 148 structures (88% of structures, 91% of the cortex) exhibit a negative trend of volume with age (Fig. 2B); of these, 37 have medium effect sizes. In UKBB males and females, the negative trend occurs across 137/148 (93% of structures, 92% of the cortex) and 118/148 (80% of structures, 83% of the cortex) structures, respectively. In UKBB males (Fig. 1C), the horizontal ramus of the right anterior lateral sulcus (a structure in the frontal lobe; ${\beta }_{UK}=-0.57$%/year) decreases in volume fastest but only the left superior frontal gyrus has a medium effect size of age for ${\beta }_{UK}$, which trends negatively at a rate of − 0.404%/year ($p$< 0.001, ${\beta }_{s}$ =  − 0.34). In UKBB females (Fig. 2C), the right angular gyrus (parietal lobe; ${\beta }_{UK}=-0.34$%/year) decreases in volume fastest but all structures exhibit only small (${\beta }_{s }<0.3$) effects of age on regional volume decrease.

**Table 2:** Brain structures exhibiting age-related trends with medium effect sizes in Tsimane and Moseten. *R*, right; *L*, left; *hemi*, hemisphere

Group	Hemi	Structure	$\beta$[%/year]	${\beta }_{S}$[unitless]
Tsimane males	L	Planum polare of the superior temporal gyrus	− 1.201	− 0.430
	R	Anterior transverse temporal gyrus (of Heschl)	− 0.961	− 0.420
Tsimane females	L	Opercular part of the inferior frontal gyrus	− 1.257	− 0.441
	L	Anterior transverse temporal gyrus (of Heschl)	− 1.056	− 0.437
	L	Planum polare of the superior temporal gyrus	− 1.231	− 0.424
Moseten males	L	Planum polare of the superior temporal gyrus	− 1.384	− 0.516
	L	Short insular gyri	− 0.777	− 0.499
	R	Anterior transverse temporal gyrus (of Heschl)	− 1.140	− 0.496
	R	Subcallosal gyrus	− 1.213	− 0.492
	L	Anterior transverse temporal gyrus (of Heschl)	− 1.368	− 0.488
	L	Long insular gyrus and central sulcus of the insula	− 0.853	− 0.482
	R	Parahippocampal gyrus	− 0.726	− 0.469
	R	Planum polare of the superior temporal gyrus	− 1.187	− 0.449
	R	Short insular gyri	− 0.716	− 0.444
	L	Gyrus rectus	− 0.866	− 0.420
	L	Subcallosal gyrus	− 0.968	− 0.403
Moseten females	R	Transverse frontopolar gyri and sulci	− 1.298	− 0.541
	L	Short insular gyri	− 0.878	− 0.522
	L	Opercular part of the inferior frontal gyrus	− 1.391	− 0.497
	L	Transverse frontopolar gyri and sulci	− 1.213	− 0.493
	L	Long insular gyrus and central sulcus of the insula	− 0.862	− 0.482
	R	Fronto-marginal gyrus (of Wernicke) and sulcus	− 1.129	− 0.476
	L	Fronto-marginal gyrus (of Wernicke) and sulcus	− 1.069	− 0.467
	R	Short insular gyri	− 0.827	− 0.457
	L	Planum polare of the superior temporal gyrus	− 1.312	− 0.422
	L	Gyrus rectus	− 0.906	− 0.419
	L	Anterior transverse temporal gyrus (of Heschl)	− 1.098	− 0.401

### T/M positive trend of occipital volumes with age

In T/M (Figs. 1A and B and 2A and B), small, but significant, positive cross-sectional trends of brain volume with age were found in occipital, posterior parietal, and posterior temporal structures, including the subparietal, medial occipito-temporal, and lingual sulci (occipital lobe). Table 3 lists structures exhibiting these trends with ${\beta }_{S}>0.10$. By comparison, in the UKBB cohort, all structures with ${\beta }_{UK}>0$ have negligible effect sizes (${\beta }_{s}<0.10$). None of the regions exhibits positive trends in all four groups (Tsimane/Moseten males/females). The right collateral and lingual sulci exhibit positive trends in Moseten males and females and in Tsimane males, while six structures exhibit this trend in the two groups. In Moseten males, 49 of 148 structures (33% of structures, 23% of the cortex) exhibit positive age-related trends in regional volumes, of which only 24 have ${\beta }_{S}>0.10$. In Moseten females, 16 of 148 structures (11% of structures, 7% of the cortex) exhibit this trend and two structures have ${\beta }_{S}>0.10$. Similarly, in Tsimane males, 25 of 148 structures (17% of structures or 12% of the cortex) exhibit a positive trend of regional volume with age (5 with ${\beta }_{s}>0.10$). In Tsimane females, 8 of 148 structures (5% of structures, 6% of the cortex), two with ${\beta }_{S}>0.10$ (Table 3), exhibit this trend.

**Table 3:** Brain structures exhibiting positive age-related trends in regional volume for Tsimane and Moseten. *R*, right; *L*, left; h*emi*, hemisphere

Group	Hemi	Region	$\beta$[%/year]	${\beta }_{S}$[unitless]
Tsimane males	L	Posterior ramus of the lateral sulcus	0.419	0.178
	R	Subparietal sulcus	0.410	0.149
	R	Marginal branch of the cingulate sulcus	0.273	0.129
	R	Calcarine sulcus	0.503	0.116
	R	Medial collateral and lingual sulci	0.285	0.107
Tsimane females	L	Subparietal sulcus	0.549	0.178
	L	Posterior ramus (or segment) of the lateral sulcus	0.338	0.136
Moseten males	R	Medial collateral and lingual sulci	0.929	0.307
	L	Intraparietal sulcus and transverse parietal sulci	1.438	0.247
	R	Anterior transverse collateral sulcus	0.804	0.242
	R	Sulcus intermedius primus	1.582	0.224
	R	Posterior transverse collateral sulcus	1.385	0.219
	L	Subparietal sulcus	0.582	0.212
	L	Parieto-occipital sulcus	0.594	0.209
	R	Parieto-occipital sulcus	0.803	0.208
	L	Sulcus intermedius primus	2.392	0.195
	L	Transverse temporal sulcus	1.628	0.193
	L	Anterior transverse collateral sulcus	0.534	0.190
	R	Intraparietal sulcus and transverse parietal sulci	0.771	0.186
	L	Anterior occipital sulcus and preoccipital notch	0.875	0.179
	R	Lateral occipito-temporal sulcus	0.403	0.175
	L	Medial collateral and lingual sulci	0.260	0.175
	L	Middle occipital sulcus and lunatus sulcus	1.552	0.145
	L	Inferior temporal sulcus	0.373	0.143
	L	Superior temporal sulcus	0.385	0.140
	L	Lateral occipito-temporal sulcus	0.247	0.128
	L	Central sulcus	0.509	0.125
	L	Posterior transverse collateral sulcus	0.364	0.124
	R	Calcarine sulcus	0.517	0.120
	L	Superior occipital and transverse occipital sulci	0.550	0.107
	R	Subparietal sulcus	0.278	0.106
Moseten females	R	Medial collateral and lingual sulci	0.741	0.232
	L	Posterior transverse collateral sulcus	0.449	0.132
	L	Lateral occipito-temporal sulcus	0.207	0.102

### Comparison of Tsimane/Moseten to UKBB participants

The annual rate of total brain volume decrease with age differs significantly across Tsimane, Moseten, and UKBB participants of either sex (${\beta }_{T}= -0.22\mathrm{\%}/y$, 95% CI = [− 0.24, − 0.19]$\mathrm{\%}/y$; ${\beta }_{M}=-0.29\mathrm{\%}/y$, 95% CI = [− 0.32, − 0.26]$\mathrm{\%}/y$; ${\beta }_{UK} = -0.18\mathrm{\%}/y$, 95% CI = [− 0.18, − 0.17]$\mathrm{\%}/y$). For each region, Supplementary material 2 provides a complete list of comparisons between groups. Supplementary Material 3 lists age-related rates of volume decrease for the entire brain, total white matter, total GM, and cortical GM in Tsimane, Moseten, and the UKBB.

#### Findings in males

Compared to T/M, UKBB participants exhibit a slightly faster age-related rate of total cortical GM volume decrease (${\beta }_{T}=-0.08\%/y, {\beta }_{M}=-0.09\%/y, {\beta }_{UK}= -0.11\%/y$). In UKBB males, the volume of more cortical GM structures trends more negatively with age than in Tsimane (Fig. 1D) or Moseten (Fig. 1E). The age-related trends of 137 of 148 structures differ significantly between the UKBB and Tsimane, with 54% of cortical volume decreasing faster in the UKBB. The average cortical GM volume change $\kappa$ (see “Methods”) is − 17.08%, indicating faster cortical GM decreases in UKBB compared to Tsimane. About 37% of the cortex decreases faster in Tsimane compared to the UKBB; the remainder (about 9%) exhibits no difference in its rate of total change. Similarly, the age-related trends of 139 of 148 structures exhibit significant differences between Moseten and UKBB, with 51% of the cortex trending more negatively in the UKBB ($\kappa = -11.82\%$). About 40% of cortical volume decreases faster in Moseten than in the UKBB. Table 4 lists structures that exhibit the steepest rates of volume decrease in the T/M compared to UKBB and Table 5 lists structures that exhibit the steepest rates of volume decrease in the UKBB compared to T/M. Many frontal and temporal structures trend significantly more negatively in UKBB males than in T/M males (blue structures in Fig. 1D, E). These include the left anterior segment of the lateral sulcus (${\beta }_{T}=-0.07\%/y, {\beta }_{M}=0.30\%/y, {\beta }_{UK}= -0.45\%/y$), the medial occipito-temporal sulcus (collateral sulcus), and the lingual sulcus (Table 5).

**Table 4:** Brain structures with the most negative t-statistics pertaining to the comparison of age-related trends between Tsimane/Moseten and UKBB. More negative t-statistics denote that the regions in question trend more negatively with age in Tsimane/Moseten than the UKBB. All tests have *p* < 0.001. *R*, right; *L*, left; *hemi*, hemisphere; *df*, degrees of freedom

Group	Hemi	Region	$\beta$[%/y]	$\sigma \left(\beta \right)$[%/y]	${\beta }_{UK}$[%/y]	$\sigma \left({\beta }_{UK}\right)$[%/y]	*T*	*df*
Tsimane males	L	Short insular gyri	− 0.61	0.16	− 0.05	0.12	− 89	1842
	L	Planum polare of the superior temporal gyrus	− 1.20	0.27	− 0.33	0.17	− 87	1667
	R	Short insular gyri	− 0.56	0.13	− 0.07	0.13	− 81	1997
	L	Opercular part of the inferior frontal gyrus	− 0.98	0.24	− 0.29	0.16	− 75	1699
	R	Anterior transverse temporal gyrus	− 0.96	0.22	− 0.30	0.18	− 72	1931
Tsimane females	L	Opercular part of the inferior frontal gyrus	− 1.26	0.25	− 0.19	0.13	− 119	1538
	L	Short insular gyri	− 0.68	0.15	0.07	0.15	− 111	1992
	L	Anterior transverse temporal gyrus	− 1.06	0.22	0.01	0.23	− 107	1992
	L	Fronto-marginal gyrus and sulcus	− 0.98	0.23	− 0.11	0.14	− 105	1650
	R	Short insular gyri	− 0.72	0.15	0.02	0.16	− 104	1992
Moseten males	L	Short insular gyri	− 0.78	0.15	− 0.05	0.12	− 122	1919
	L	Planum polare of the superior temporal gyrus	− 1.38	0.23	− 0.33	0.17	− 117	1825
	R	Subcallosal gyrus	− 1.21	0.23	− 0.03	0.24	− 113	1994
	R	Short insular gyri	− 0.72	0.14	− 0.07	0.13	− 103	1988
	R	Planum polare of the superior temporal gyrus	− 1.19	0.25	− 0.23	0.16	− 101	1720
Moseten females	L	Short insular gyri	− 0.88	0.14	0.07	0.15	− 149	1992
	L	Opercular part of the inferior frontal gyrus	− 1.39	0.24	− 0.19	0.13	− 139	1587
	R	Transverse frontopolar gyri and sulci	− 1.30	0.23	− 0.12	0.15	− 133	1745
	L	Transverse frontopolar gyri and sulci	− 1.21	0.21	− 0.14	0.17	− 127	1900
	L	Fronto-marginal gyrus and sulcus	− 1.07	0.21	− 0.11	0.14	− 120	1711

**Table 5:** Brain structures whose cross-sectional trend with age differs most in Tsimane/Moseten from the UKBB. A larger t-statistic magnitude implies a larger difference between Tsimane/Moseten and the UKBB in the age-related trend of the structure’s volume. All tests have *p* < 0.001. *R*, right; *L*, left; *hemi*, hemisphere; *df*, degrees of freedom

Group	Hemi	Region	$\beta$[%/y]	$\sigma \left(\beta \right)$[%/y]	${\beta }_{UK}$[%/y]	$\sigma \left({\beta }_{UK}\right)$[%/y]	*T*	*df*
Tsimane males	R	Subparietal sulcus	0.41	0.29	− 0.37	0.18	72	1663
	R	Medial collateral and lingual sulci	0.28	0.27	− 0.31	0.13	61	1422
	L	Medial collateral and lingual sulci	0.10	0.16	− 0.30	0.13	60	1882
	L	Middle-anterior part of the cingulate gyrus and sulcus	0.00	0.18	− 0.48	0.20	58	1977
	L	Subparietal sulcus	0.23	0.25	− 0.30	0.18	53	1803
Tsimane females	L	Subparietal sulcus	0.55	0.32	− 0.11	0.20	55	1672
	R	Middle temporal gyrus	0.08	0.31	− 0.26	0.11	32	1270
	L	Occipital pole	0.81	1.13	− 0.11	0.17	25	1044
	L	Inferior occipital gyrus and sulcus	0.10	0.35	− 0.19	0.19	23	1559
	R	Subparietal sulcus	0.03	0.29	− 0.20	0.19	20	1731
Moseten males	R	Medial collateral and lingual sulci	0.93	0.32	− 0.31	0.13	114	1324
	L	Intraparietal and transverse parietal sulci	1.44	0.55	− 0.16	0.14	88	1129
	L	Medial collateral and lingual sulci	0.26	0.16	− 0.30	0.13	87	1907
	L	Subparietal sulcus	0.58	0.27	− 0.30	0.18	86	1751
	R	Posterior transverse collateral sulcus	1.38	0.61	− 0.41	0.28	84	1387
Moseten females	R	Medial collateral and lingual sulci	0.74	0.32	− 0.24	0.13	90	1325
	L	Posterior transverse collateral sulcus	0.45	0.33	− 0.17	0.25	46	1872
	L	Medial collateral and lingual sulci	0.08	0.15	− 0.18	0.13	41	1958
	L	Intraparietal and transverse parietal sulci	0.37	0.51	− 0.12	0.15	29	1163
	L	Anterior transverse collateral sulcus	0.26	0.28	− 0.06	0.19	29	1748

#### Findings in females

**Fig. 3:**
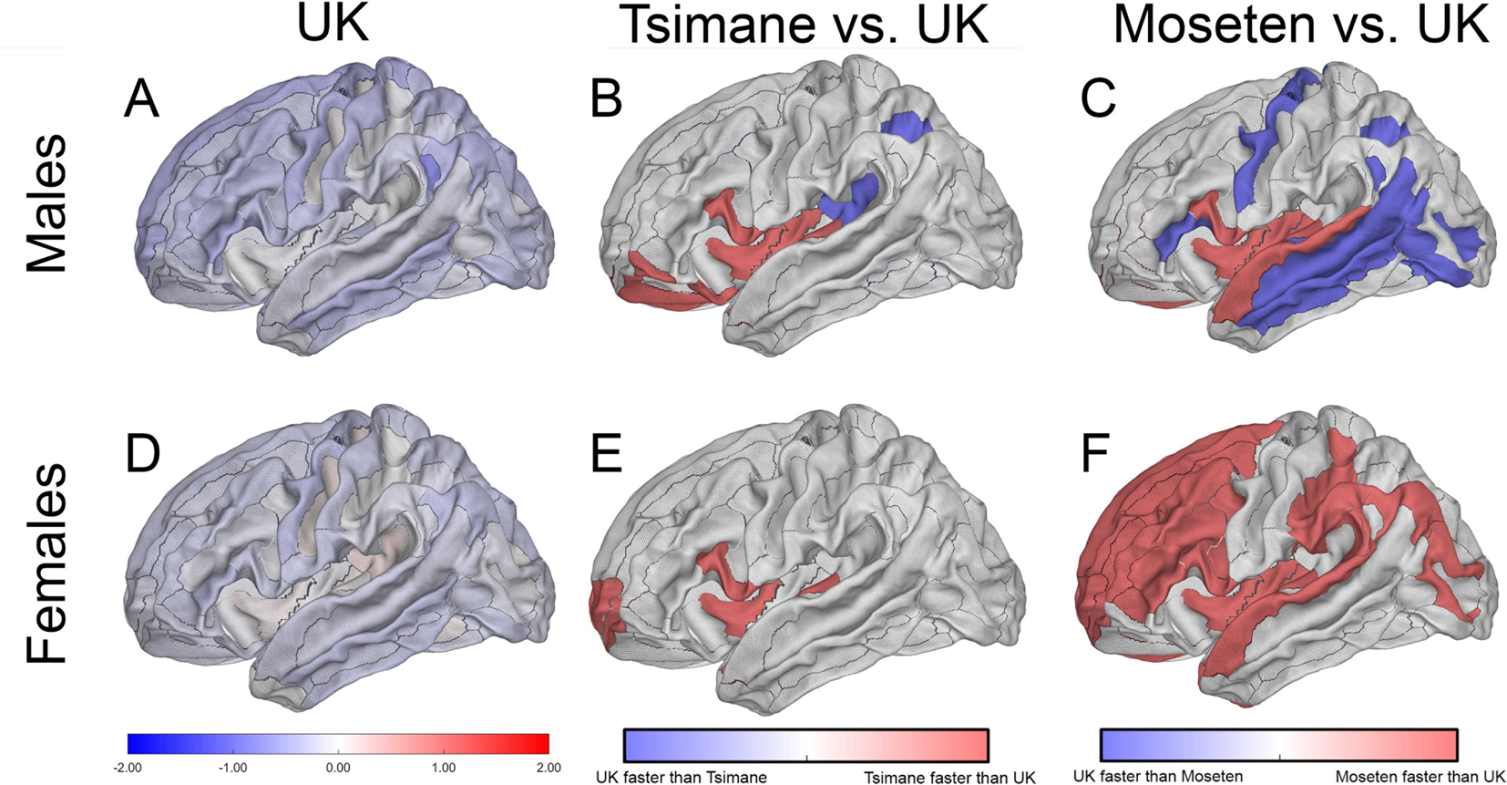
**A**, **D** Regression coefficients for the association between regional cortical volume and age in males (**A**) and females (**D**) for the UK Biobank. Brighter color indicates a greater magnitude of the regression coefficient for the corresponding structures. **B**, **C** Structures whose regression coefficients differ significantly (absolute value of *t *> 40) between (**B**) UKBB males and Tsimane males, or between (**C**) UKBB males and Moseten males. **E**, **F** Structures whose regression coefficients differ significantly (absolute value of *t* > 60) between the UKBB females and (**E**) Tsimane females or (**F**) Moseten females. Blue indicates faster volume decrease in the UKBB compared to T&M. Red indicates faster volume decrease in T&M compared to the UKBB. All plots illustrate the lateral view of the left hemisphere

Compared to the T/M, UKBB participants exhibit a slower age-related rate of total cortical GM volume decrease (${\beta }_{T}=-0.18\%/y,{\beta }_{M}=-0.20\%/y, {\beta }_{UK}= -0.06\%/y$). In Tsimane (Fig. 2D) and Moseten (Fig. 2E) females, cortical GM trends more negatively with age than in UKBB females. Structures drawn in red in Fig. 2D and E exhibit faster volume decreases in the T/M compared to UKBB. The rate of volumetric change differs significantly between Tsimane and UKBB for 140 structures ($\kappa =69.90\%$), and 82% of cortical volume decreases faster with age in Tsimane than in the UKBB. About 12% of cortical volume decreases faster with age in the UKBB compared to Tsimane. The left opercular part of the inferior frontal gyrus exhibits the largest difference between Tsimane females to their UKBB counterparts (Fig. 3E, F, Table 4). The rate of volumetric change differs significantly between Moseten and the UKBB for 141 structures ($\kappa =73.02\%$), and 84% of cortical volume decreases faster with age in Moseten compared to the UKBB. About 11% of cortical volume decreases faster in the UKBB compared to Moseten. The left short insular gyrus exhibits the largest difference between Moseten and UKBB (Table 4). Structures whose volumes decrease faster in the UKBB than in Moseten include parietal structures such as the left intraparietal and transverse parietal sulci.

### Mediation effect of visual spatial ability and physical activity

For the total volume of several structures of interest, we quantified the mediation effect for a measure of visual spatial ability (the stick design score) on these structures’ age-related total volume trend. This volume was calculated by adding the volumes of structures with positive trends in volume and with effect sizes above 0.10 within each group. Positive trends of regional brain volume with age were observed in occipital, posterior parietal, and posterior temporal regions (Table 3). This yielded ${\beta }_{a}$ (direct effect of age on regional brain volume) and ${\beta }^{\, \prime}_{a}$ (effect of age on regional volumes after controlling for the stick test score) for each sex and population. In Tsimane males, the stick test score mediated age dependence for the total volume of interest. Thus, ${\beta }_{a }=0.18$ decreases to ${\beta }^{\, \prime}_{a}=0.16$ when accounting for the indirect effect of age on volume, mediated through the visuospatial score (${t}_{193}= 1.33$, $p=0.043$). This indicates partial mediation of the relationship between age and parietal/occipital volume by the stick test score. The stick test score does not change ${\beta }_{a}$ in Tsimane females or either sex in Moseten ($p >0.05$). Physical activity (minutes of moderate activity per day) does not mediate the relationship between age and the volume of combined brain structures for either sex in the T/M ($p >0.05$). Supplementary Material 4 provides results for the mediation analysis. Supplementary Material 5 lists parameters for the distributions of daily minutes of moderate activity and stick test scores for T/M.

### Laterality effects

In all three populations, the number of structures in the left hemisphere that exhibit negative trends of volumetric change is comparable to that in the right hemisphere. This is also the case for structures with positive trends of volumetric change. For Tsimane males, 55 of 74 structures exhibit negative age-related volumetric trends in the left hemisphere and 56 of 74 structures exhibit such trends in the right hemisphere. For Tsimane females, 65 of 74 structures exhibit negative age-related volumetric trends in the left hemisphere and 69 of 74 structures exhibit such trends in the right hemisphere. Similarly, Moseten males (left = 49/74, right = 40/74), UKBB males (left = 68/74, right = 69/74), Moseten females (left = 64/74, right = 66/74), and UKBB females (left = 60/74, right = 58/74) also exhibit negative age-related volumetric trend in similar number of structures across the hemispheres. For Tsimane males, positive age-related volumetric trends were observed in 13 of 74 structures in the left hemisphere and in 12 of 74 structures in the right hemisphere. Moseten males (left = 23/74, right = 26/74), Tsimane females (left = 6/74, right = 2/74), and Moseten females (left = 8/74, right = 8/74) also exhibit positive age-related volumetric trends in similar numbers of structures across the hemispheres.

### Allometric corrections and mortality hazard

Correcting for intracranial volume allometry (Supplementary Methods) did not significantly modify any brain structure’s trend of volume with age (Supplementary Results). For these reasons, allometric corrections were not included in any regression. Cox proportional hazard models (Supplementary Methods) suggested that the age-related trends of regional volumes had a negligible effect from selective T/M mortality (Supplementary Results). Thus, the findings of the study are unlikely to be related, in a statistical sense, to the higher mortality rates of T/M.

## Discussion

### Positive cross-sectional trends of regional volume with age

In industrialized populations, across most brain structures, regional brain volume typically declines with age [2, 16–18]. In T/M males, although total brain volume decreases with age, the heterogeneity in regional trends uncovered in this work highlights the fact that the age-related rate of total brain volume decrease is a sum over both negative and positive rates of age-related *regional* volumetric changes. In other words, regional rates of both signs add up to an overall negative trend in total brain volume with age, as observed by Irimia et al. [23]. This global trend is negative because there are more structures with negative trends than structures with positive trends. Surprisingly and notably, in T/M males, a small, but significant, cross-sectional positive trend of brain volume with age is observed in occipital and parietal structures (Fig. 1A, B) implicated in spatial navigation [48, 49] and visual processing. In Tsimane males, the statistical effects of this positive trend are partially explained by visuospatial performance on the stick design test. This is because, in regression, accounting for (A) the *indirect* effect of age on volumes mediated through stick score test decreases (B) the *direct* effect of age on the volumes of structures with positive age-related volume trends (Table 3). This result suggests that the visuospatial ability quantified by this test partially explains the positive age-related trend in the volumes of these structures (Supplementary Material 4). Our interpretation is supported by Wenger et al. [50], who studied a small sample of healthy German young men. These authors observed cortical thickening in structures involved in the neural network underlying complex spatial navigation. In another study, Tseng et al. [51] found that intensive lifelong aerobic training may attenuate aging-related brain tissue loss in regions associated with visuospatial function and motor control.

In industrialized populations, the occipital lobe does not atrophy as fast as other cerebral lobes [52–54]. Nevertheless, hardly any reports of positive cross-sectional trends of brain volume with age have been reported. A cross-sectional study [55] assessing the effects of amyloid-$\beta$ and tau on cortical thickness in cognitively normal Americans found cortical thickening in the entorhinal and posterior cingulate cortex, as well as volume increase in the right insula. However, these authors used the Desikan-Killiany atlas for segmentation, whereas we used the Destrieux atlas. The latter includes the entorhinal region in the parahippocampal gyrus, and cingulate cortex is divided into three structures, none of which exhibits a positive age-related trend. Corroborating Hojjati et al. [55], the right insular structures exhibit small (negligible effect sizes, ${\beta }_{s}<0.08$) positive age-related regional volume trends in UKBB females (Fig. 2C). However, none of the other groups exhibits this trend. In a very small sample ($N<10$) of indigenous Australians, the volume of visual cortex may be preserved in comparison to Caucasian Australians, potentially reflecting the former’s adaptation to living in forests and deserts [56]. The same may be the case in T/M, who live in densely forested areas where they rely on complex visual cues to navigate and subsist [27, 57]. Previous studies among the Tsimane report little or no decline in route-finding ability with age [48, 49], nor are there sex differences in dead-reckoning ability. While men engage in more hunting than women and have greater travel distances [49, 58, 59], both sexes travel extensively in a dense forest environment with few visuospatial aids such as mountains. Consistent with this observation, structures such as the subparietal sulcus (Fig. 1 A, B, Table 3)—which is involved in memory recall, visual scene processing, and navigation [60]—exhibit a positive age-related trend of volume in T/M.

### Physical activity and cognition

In older adults, cognitive health can improve by adjusting diet and increasing physical activity. Even low-to-moderate intensity activities (e.g., household chores) can slow the progression of age-related cognitive decline [61, 62]. Furthermore, a multi-domain intervention incorporating physical activity, diet, and cognitive training can result in significant cognitive improvements [63]. Studies of older adults in industrialized countries, including the US, suggest that physical activity can decelerate brain atrophy and perhaps even lead to regional increases in cortical GM [64–68]. For example, in one study of older adults, 6 months of aerobic fitness training was associated with increases in GM and white matter volumes [66]. In rats, aerobic exercise can lead to the production of growth hormones such as brain-derived neurotrophic factor [69, 70] and insulin-like growth factor [71, 72]. These hormones can facilitate the creation of capillaries, the synthesis of dendritic connections, and the birth of cells in the hippocampus [73–75]. For Tsimane, total energy expenditure is 264 kcal/day higher than in industrialized populations [76], especially for males [77]. Thus, because T/M engage in significantly more physical activity than most persons in industrialized countries [48, 76], the positive cross-sectional trend of regional brain volume with age reported here may be due partly to high levels of physical activity. However, we found no evidence that moderate physical activity mediates the relationship between age and brain volume. This may be due to a lack of power, as accelerometry data were only available for 67% of participants. In addition, accelerometry data were collected on average 3.8 years after CT scans, and brain volume trends with age may accelerate or decelerate over time. Thus, a lack of mediation effect may be due to poor temporal correspondence between accelerometer and CT measurements. It should be mentioned that typical mediation analysis examines how volume loss leads to cognitive decline. Our main regression, however, examines how age leads to volume loss, so it is more straightforward conceptually to undertake a mediation analysis seeking to understand whether cognitive decline mediates that relationship. Furthermore, this type of mediation analysis highlights how greater use of a cognitive function can act on brain volume by helping to prevent its loss. Whereas studying the effect of volume loss on cognitive decline is important, the examination of this effect is outside the scope of the present study.

### Comparison of Tsimane/Moseten to the UKBB

The Tsimane population studied here exhibits age-related cross-sectional rate of total brain volume decrease of ${\beta }_{T}=-0.22\%/y$, corroborating Irimia et al. [23] and Kaplan et al. [24]. The rate of decrease for the Tsimane is slower than in the reference population from the Netherlands ($\beta =$ −0.37%/y for 5286 Dutch participants aged 45 to 95) studied by Irimia et al. [23]. Irimia et al. [23] and Kaplan et al. [24] studied total brain volume change but, as Figs. 1, 2, and 3 illustrate, the regional rate of volumetric change varies across brain structures, sexes, and populations. Furthermore, our sample of 19,973 UKBB participants (${\beta }_{UK}=$ −0.18%/y, Supplementary Material 3) exhibits a slower age-related rate of total brain volume change compared to the Tsimane and to two populations from the US or Germany studied previously. This result should be interpreted carefully (see Supplementary Discussion).

This study compares age-related rate of regional cortical gray matter (GM) volumes on age in T/M to UKBB participants according to sex. The negative $\kappa$ of T/M indicates that, on average, UKBB males’ cortex decreases in volume faster than T/M. This is corroborated by a slightly faster rate of total cortical GM volume decrease in UKBB males compared to T/M (Supplementary Material 3). Furthermore, the positive $\kappa$ of T/M females indicates that their total cortical GM trends negatively with age *faster* than in sex- and age-matched UKBB participants (Fig. 2D, E). UKBB females’ slower rate of total cortical GM volume decrease compared to T/M supports these trends. In T/M females, 83% of cortical volume decreases with age faster than in UKBB females. Regional differences in the trend of brain volume with age may involve sex differences in hormones, lifestyle, health trajectories [5, 23, 78–80], or fertility [81, 82]. In addition to higher obesity rates for the Tsimane females than the males [83], there are substantial differences in the daily activities of males and females. T/M females spend more time caring for children (breastfeeding, grooming), preparing food, and engaging in light physical activity. Males, especially those under 60, engage in more moderate to vigorous physical activity outside the home, such as fishing, hunting, and farming.

In industrialized populations, the frontal and temporal lobes, striatum, cerebellum, and hippocampus atrophy faster than the rest of the brain in both sexes [19]. Frontal and temporal structures atrophy fastest in males, whereas the hippocampi and parietal lobes atrophy fastest in females [4, 20]. In agreement with these studies, UKBB males exhibit faster atrophy in the horizontal ramus of the anterior right lateral sulcus (frontal lobe) compared to other structures. UKBB females exhibit faster atrophy in the right angular gyrus (parietal lobe). Tsimane males’ *regional* rates of GM volume decrease with age are slower than in UKBB males. By contrast, T/M females exhibit a faster cross-sectional regional volume trend with age compared to UKBB females. In Moseten females, around 85% of the cortex exhibits faster age-related decline in regional brain volume compared to UKBB females (Fig. 2E); only 40% of the cortex atrophies faster in Moseten males compared to UKBB males (Fig. 1E). As Fig. 3E and F illustrates, cortical structures such as the left short insular gyrus and the opercular part of the left inferior frontal gyrus (Table 4) atrophy significantly faster in T/M females compared to their industrialized counterparts.

No lateralization effect on age associations was observed in Tsimane, Moseten, or UKBB. As Figs. 1 and 2A–C illustrate, a similar number of structures in either hemisphere exhibit age-related volumetric change in these populations. When comparing Tsimane males to UKBB males (left hemisphere: $\kappa =-8\%$, right hemisphere: $\kappa =-9\%$, Fig. 1D), a similar mean amount of cortex exhibits cross-sectional decrease with age in both hemispheres. Similarly, for the comparison of Moseten males to UKBB males, $\kappa =-6\%$ for both hemispheres (Fig. 1E). When comparing Tsimane females to UKBB females (left hemisphere: $\kappa =31\%$, right hemisphere: $\kappa =39\%,$ Fig. 2D), cortex in the right hemisphere exhibits more cross-sectional decrease with age in the Tsimane. However, when comparing Moseten females to UKBB females (left hemisphere: $\kappa =39\%$, right hemisphere: $\kappa =34\%$, Fig. 2E), cortex in the left hemisphere exhibits more cross-sectional decrease with age in the Moseten.

### Regions with steeper age-related change in brain volume for Tsimane/Moseten

T/M exhibit cross-sectional volumetric decreases with age that are faster for the inferior frontal gyri (Table 2) than for most other structures. In industrialized populations, faster cross-sectional decreases in the volumes of frontal structures with age may reflect diminished higher-order cognitive functions such as semantic and working memory, impulse control and inhibition, speech production and phonological processing, planning, and sensory integration [84–86]. It is possible that higher-order cognitive functions in T/M may also decrease, similarly to industrialized populations. However, further psychometric testing and analyses are required to clarify the functional significance of these findings.

Similarly to industrialized populations [17, 87–89], the left superior temporal gyrus and both anterior transverse temporal (Heschl’s) gyri exhibit faster cross-sectional decrease with age, in T/M, compared to other structures (Figs. 1 and 2A, B, Table 2). The left superior temporal gyrus is involved in speech perception and production [90]. The short insular gyri exhibit faster cross-sectional decrease with age compared to other structures in T/M. The insulae are linked to audio-visual integration tasks [91, 92], consciousness, emotion regulation [93], and homeostasis [94]. Ultimately, it may be difficult to measure cognitive functions in ways that are culturally invariant across the Tsimane/Moseten and US/EU. For this reason, exploring how the relationship between regional brain volume changes and cognitive functions differs between T/M and industrialized populations is challenging, particularly as T/M undergo societal changes.

### Limitations

In this cross-sectional design, atrophy was not measured directly. For this reason, the decrease in regional brain volume with age was calculated as the annual percentage change in regional volume. Although repeated measures within subjects allow direct measurements of volume change, this cross-sectional study provides a foundation for future longitudinal studies. Comparisons between industrialized and non-industrialized populations as a function of age could reveal further information as disease risk increases. Epigenetic factors and genetic makeup may provide additional insights into brain volume change in the studied populations. Even though our bootstrapping reduced bias introduced by differences in samples sizes, findings may be affected by the smaller T/M sample size compared to the UKBB. Ultimately, this smaller sample size is constrained by the small size of the T/M population relative to that of the UK. The UKBB is not representative of the UK population because of healthier volunteer selection bias [39]. Furthermore, this study does not quantify industrialization. Nevertheless, the UKBB’s large size and geographic coverage provide a setting for comparing T/M to individuals in an industrialized society. Finally, the comparison of T/M to UKBB participants may be confounded, in part, by the fact that T/M volumetrics were derived from CT, whereas UKBB volumetrics were extracted from MRI. This is because MRI was not available in the remote geographical region of Bolivia inhabited by the former two populations. Nevertheless, we do not expect the effect of this confound to be substantial partly because (A) the regional volumes derived from CT and MRI differ by 2.5% on average and (B) cross-sectional trends of volume with age were computed within each modality.

## Conclusion

Lifestyle factors can influence the population-level rate of regional brain volume decrease with age. On average, compared to UKBB participants, T/M males exhibit slower rates of regional volume decrease—or, indeed, even positive cross-sectional trends of brain volume with age—in some brain structures. Whereas the UKBB, on average, exhibit faster volume decreases in frontal and temporal structures, the same brain structures experience significantly slower decreases in volume with age in T/M. Notably, females exhibit faster atrophy than their UKBB counterparts. This highlights the putatively protective effects of a non-industrial lifestyle in Tsimane/Moseten males, but not in females (who are at higher risk for Alzheimer’s disease in industrialized countries). Alternatively, factors associated with industrialization that are unknown to us may attenuate females’ rate of regional volume decrease with age. In the light of (epi)genetic effects, future research is warranted to elucidate the associations of these effects with age-related regional volume trends physical activity, diet, neurodegenerative disease risk, and cognitive functioning.

### Supplementary Information

Below is the link to the electronic supplementary material.

Supplementary file1 This workbook provides a complete list of regression coefficients and effect sizes representing rate of regional brain volume change with age. Each sheet is named based on the group. For example: "MaleTsi" contains regression coefficients and effect sizes along with their standard deviations for the Tsimane males. (XLSX 151 KB)

Supplementary file2 This workbook provides a list of group-wise comparisons of regression coefficients representing age-related brain volumes for each cortical structure in the human brain. Sheet names denote the groups compared. (XLSX 135 KB)

Supplementary file3 Regression coefficients for age-related tissue level volume change for Tsimane, Moseten and UKBB. Sheets names corresponds to different sexes. (XLSX 15 KB)

Supplementary file4 This workbook contains results for the mediation analysis. The sheets correspond to each of the mediating variables: the visuospatial ability (stick Design Test), and physical activity (moderate Minutes). (XLSX 16 KB)

Supplementary file5 This workbook lists parameters for the distributions of daily minutes of moderate activity and stick test scores for Tsimane and Moseten distribution of mediating variables for the groups. The name of sheets correspond to each of the mediating variables. (XLSX 3340 KB)

Supplementary file6 This workbook contains result from the Cox hazards analysis to quantify the effect of sex, age, and regional brain volumes on mortality for the Tsimane and Moseten. The sheets are named based on the groups. (XLSX 57 KB)

Supplementary file7 (DOCX 51 KB)

### Supplementary Information

Below is the link to the electronic supplementary material.

## Data Availability

Individual-level data for the Tsimane and Moseten are stored in the Tsimane Health and Life History Project (THLHP) data repository. THLHP data access is restricted due to ethical reasons. To request individual-level data, an application must be submitted that includes specific details regarding the intended use of the data, research questions to be addressed, procedures for data security and individual privacy, potential benefits to the study communities, and methods for assessing and minimizing stigmatizing interpretations of the research outcomes. Data sharing policy and request forms are available at https://tsimane.anth.ucsb.edu/data.html). Requests for individual-level data will require institutional IRB approval (even if exempt) and will be reviewed by an Advisory Council composed of tribal leaders, tribal community members, Bolivian scientists, and the THLHP leadership. A similar structure exists for the Moseten data.

## Abbreviations

CT: Computed tomography
FS: FreeSurfer
GM: Gray matter
MRI: Magnetic resonance imaging
T/M: Tsimane and Moseten
UKBB: UK BioBank
