# Supplementary material for: Increases in regional brain volume across two native South American male populations
Source: GeroScience. 2024 Apr 29;46(5):4563–83. doi: 10.1007/s11357-024-01168-2 (PMC11336037; doi:10.1007/s11357-024-01168-2)
Supplement: Supplementary file 7 — Supplementary file7 (DOCX 51 KB) [file 11357_2024_1168_MOESM7_ESM.docx]

All following supplementary sections are intended for publication as an online data supplement.

Supplementary Methods

Because brain volume can decrease nonlinearly with age, we explored the utility of a nonlinear allometric correction to brain volumes. To this end, for each structure, in T/M, we used a sex-specific log-log regression to estimate the significance of the nonlinear relationship between ICV and regional volume.

For each brain structure (N = 148), we evaluated the potential effect of mortality selection on the observed relationship between age and volume in the T/M. Cox proportional hazard models were used to quantify the effect of sex, age, and regional brain volumes (independent variables) on mortality (dependent variable) for the T/M. The Cox proportional hazard coefficients indicate whether risk of death trends significantly with each of the independent variables. For example, a positive coefficient for the effect of age on mortality would indicate that older participants have a higher risk of death than younger participants do. Similarly, a positive coefficient for a regional brain volume would indicate that larger volume of that region is associated with greater risk of mortality. The Cox proportional hazard coefficients were statistically tested for significance and the $p$-values were corrected for multiple comparisons using Bonferroni corrections with $\alpha= 0.05/148$.

Supplementary Results

Across 70% of brain structures, the coefficients of determination ($R^{2}$) for the log-log regression models estimating allometric coefficients were below 0.2, indicating that under 20% of ICV variance was explained by regional brain volume. Correcting for intracranial volume allometry did not significantly modify any brain structure’s trend of volume with age. For these reasons, allometric corrections were not included in any regression.

In T/M, Cox proportional hazard models were used to quantify the effect of sex, age, and regional brain volumes (independent variables) on mortality (dependent variable) for the T/M. The Cox proportional hazard coefficients indicated whether risk of death trends significantly with each of the independent variables. In T/M, the Cox proportional hazard coefficients of age were positive for all brain structures. The log hazard model coefficients indicate whether death risk trends significantly with other independent variables. For this reason, our finding of positive coefficients for age suggests, that older T/M participants have higher risk of death than younger participants, as expected. None of the Cox proportional hazard coefficients of sex or regional volumes (**Supplementary material 6**) were statistically significant after multiple comparisons correction. This suggests that the age-related trends of regional volumes had a negligible effect from selective T/M mortality (see *Methods*). Thus, the findings of the study are unlikely to be related, in a statistical sense, to the higher mortality rates of T/M.

Supplementary Discussion

Differences between our findings and those of Irimia, Chaudhari [23] should be interpreted carefully for several reasons. Firstly, the UKBB sample is not representative of the UK population; for example, its average participant is healthier and less likely to be socioeconomically disadvantaged than the average UK person [38]. Perhaps, for this reason, in the UKBB, the change in brain volume with age is shallower than in other population samples. Thus, the results suggest that healthy individuals in industrialized populations have rates of brain volume loss with age that are as shallow or shallower than the physically active native Bolivian populations. Secondly, the present comparison of Tsimane to UKBB participants involves genuine brain volumes computed from participants’ MRIs. By contrast, the individual brain volumes of participants from Germany, the US, and the Netherlands were unavailable to Irimia, Chaudhari [23]. These authors’ comparison involved the imputation of age-related volume trends from simulations that made assumptions about volume distributions and statistics. Thirdly, although our UKBB sample size ($N$ = 19,973) is larger than those considered by Irimia et al. (Netherlands: $N$ = 5,286; US: $N$ = 316; Germany: $N$ = 248), post-industrialized populations may exhibit variability in brain volume trajectories that was not fully captured in this study. These considerations highlight (A) differences in sampling and subsequent bias across samples, (B) methodological differences between studies, (C) technical limitations of the previous study, and (D) the variability of total brain volume trajectories within industrialized populations. Conclusions involving the comparison of industrialized cohorts to the Tsimane are likely to depend on characteristics of the sample chosen.
